# Multi-Sequence Guided Generation of Contrast-Enhanced Magnetic Resonance Imaging Using Diffusion Models

**DOI:** 10.3390/bioengineering13060634

**Published:** 2026-05-28

**Authors:** Yue Xu, Xiaokun Zhou, Wei Jiang, Chuanbing Wang, Xiangnan Geng, Da Cao, Wujin Xiao, Bin Liu, Wei Wang

**Affiliations:** 1 Department of Clinical Medical Engineering, The First Affiliated Hospital of Nanjing Medical University, Jiangsu Province Hospital, Nanjing 210029, China; xuyue@jsph.org.cn (Y.X.); geng0703255@126.com (X.G.); xwj6863@163.com (W.X.); 2 Engineering Research Center of Intelligent Theranostics Technology and Instruments, Ministry of Education, School of Biomedical Engineering and Information, Nanjing Medical University, Nanjing 211166, China; bmezxk@stu.njmu.edu.cn; 3 Department of Radiology, The First Affiliated Hospital of Nanjing Medical University, Jiangsu Province Hospital, Nanjing 210029, China; jiangweijsph@163.com (W.J.); wangchuanb_csr@163.com (C.W.); yyzzcaoda@163.com (D.C.)

**Keywords:** contrast-enhanced magnetic resonance imaging, image generation, diffusion model, ControlNet, difference-aware guidance

## Abstract

**Objectives**: Contrast-enhanced magnetic resonance imaging (CE-MRI) plays an important role in the diagnosis, treatment monitoring, and follow-up of brain tumors. However, the use of gadolinium-based contrast agents (GBCAs) is limited in patients with contraindications, such as severe renal impairment or situations requiring repeated examinations. This study aimed to develop a diffusion model-based Difference-Aware Guided Control Network (DAGCN) for synthesizing high-quality contrast-enhanced T1-weighted MRI (T1-CE) from non-contrast T1-weighted images in combination with an auxiliary sequence. **Methods**: Using the BraTS 2021 dataset, we proposed a two-stage generative framework that first localizes lesion-related enhancement cues and then guides image synthesis. In the first stage, a Difference-Aware Fusion and Prediction (DAFP) module was designed to extract complementary information from non-contrast T1-weighted images and an auxiliary sequence (T2-weighted or FLAIR) through dual-branch feature extraction and cross-modal channel attention fusion, followed by prediction of a lesion-related discrepancy map. In the second stage, the predicted discrepancy map was concatenated with the original T1-weighted images and introduced into a ControlNet-guided diffusion model to constrain the reverse denoising process and generate the target T1-CE image. Model performance was evaluated by visual comparison, quantitative metrics including peak signal-to-noise ratio (PSNR), structural similarity index measure (SSIM), visual information fidelity (VIF), and normalized cross-correlation (NCC), as well as blinded radiologist scoring of image quality (IQ), clinical replaceability (IC), contrast enhancement (CE), and lesion conformity (CF). **Results**: DAGCN generated synthetic T1-CE images with preserved global anatomical structure and faithful local lesion enhancement without the need for contrast agent administration. Compared with baseline methods, DAGCN achieved the highest PSNR and NCC under both T1 + T2 and T1 + FLAIR settings, while showing competitive SSIM and VIF performance. Visual comparison and radiologist-based subjective evaluation further indicated improved lesion-focused enhancement fidelity and reduced false-positive enhancement. Among the two auxiliary sequence settings, the T1 + FLAIR configuration provided more specific lesion localization and cleaner background suppression than the T1 + T2 configuration, particularly by reducing interference from cerebrospinal fluid signals. **Conclusions**: The proposed DAGCN framework enables the synthesis of clinically informative contrast-enhanced-like MRI from non-contrast multi-sequence inputs and may provide a promising alternative for patients in whom gadolinium administration is contraindicated or should be avoided. In particular, the FLAIR-guided setting showed advantages in lesion specificity, background cleanliness, and overall diagnostic quality.

## 1. Introduction

In clinical neuroimaging, contrast-enhanced magnetic resonance imaging (CE-MRI) is essential for the diagnosis, treatment monitoring, and longitudinal follow-up of brain tumors and other neurological diseases because of its excellent soft-tissue contrast and lesion conspicuity [1,2]. Gadolinium-based contrast agents (GBCAs) shorten T1 relaxation time and thereby increase lesion conspicuity, making them a routine component of many neuroimaging protocols [3,4,5].

However, increasing evidence has raised concerns regarding GBCA safety. Even in patients with normal renal function, gadolinium deposition has been reported in the brain, particularly in the dentate nucleus and globus pallidus, and the extent of deposition appears to increase with repeated contrast-enhanced examinations [6,7]. Such deposition has been associated with potential neurotoxicity [8], and GBCAs may also trigger nephrogenic systemic fibrosis in patients with severe renal insufficiency [9,10]. Consequently, reducing or avoiding contrast administration while preserving diagnostic image quality has become an important clinical and technical goal [10].

Recent AI-based studies have demonstrated the potential of deep learning frameworks for MRI-based brain tumor detection, classification, and lesion analysis, further supporting the feasibility of data-driven approaches in neuro-oncological imaging [11,12].

To address these concerns, increasing attention has been given to contrast agent dose reduction and contrast-free CE-MRI synthesis [13,14,15,16]. Existing approaches mainly rely on convolutional neural networks or other deep learning image-to-image translation models to synthesize CE-MRI from non-contrast or low-dose MR images [17,18]. Without explicit guidance on lesion-related enhancement, however, these models may still suffer from insufficient local enhancement, missed subtle enhancing lesions, local intensity discrepancies, or limited interchangeability with full-dose contrast-enhanced images [19,20,21,22].

Despite recent progress in artificial contrast and cross-modality MRI synthesis, several research gaps remain. First, many existing CE-MRI synthesis methods rely mainly on direct image-to-image mapping and do not explicitly localize where contrast enhancement should occur. Second, false-positive enhancement and missed subtle enhancement remain clinically important risks when the generation process is weakly constrained. Third, comparisons with modern medical image synthesis or diffusion-based generative baselines, together with statistical significance and efficiency analyses, are still insufficiently reported for this task.

To address these gaps, we developed a diffusion-based Difference-Aware Guided Control Network (DAGCN) as the proposed framework of this study. Building on diffusion-model principles [23] and ControlNet-based spatial conditioning [24], DAGCN makes the following contributions: (1) We propose a difference-aware guided diffusion framework for synthesizing T1-CE images from non-contrast multi-sequence MRI. (2) We introduce a Difference-Aware Fusion and Prediction module to explicitly estimate lesion-related enhancement cues before image generation. (3) We integrate the predicted discrepancy map into a ControlNet-guided diffusion model to spatially constrain enhancement synthesis. (4) We systematically compare T2- and FLAIR-guided synthesis using objective metrics, radiologist-based scoring, statistical testing, and computational-efficiency analysis.

The remainder of this paper is organized as follows: Section 2 describes the proposed framework and experimental design; Section 3 reports quantitative, qualitative, statistical, and efficiency results; Section 4 discusses the findings, limitations, and future directions; and Section 5 concludes the study.

## 2. Materials and Methods

### 2.1. Overview of the Difference-Aware Guided Control Network

**Figure 1:**
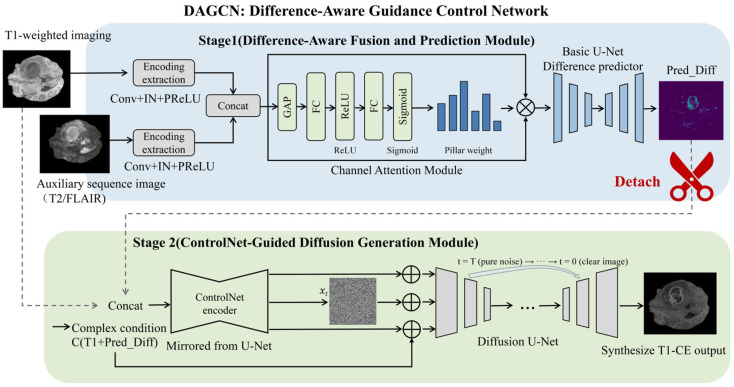
Overall architecture of the proposed DAGCN. The framework adopts a two-stage cascaded design consisting of a Difference-Aware Fusion and Prediction (DAFP) module and a ControlNet-guided diffusion generation module. The DAFP module predicts a lesion-related discrepancy map from non-contrast T1 and an auxiliary sequence (T2 or FLAIR), and the predicted discrepancy map is then concatenated with T1 to guide diffusion-based synthesis of the target T1-CE image.

As illustrated in Figure 1, DAGCN adopts a strongly decoupled two-stage cascade design. The overall framework consists of two core components: (1) a Difference-Aware Fusion and Prediction Module for identifying likely enhancement-related regions, and (2) a ControlNet-guided diffusion generation module for synthesizing the final T1-CE image under explicit spatial guidance.

Difference-aware feature prediction stage: The network first receives paired T1-weighted sequences and selected auxiliary sequences (T2 or FLAIR) as input. To fully utilize the anatomical and specific pathological boundary information provided by both sequences, this module integrates dual-sequence features through a channel attention fusion mechanism and inputs them into a difference predictor (based on BasicU-Net architecture) to predict the “discrepancy map” (i.e., enhancement increment) between non-contrast and enhanced images [25,26]. The output of this stage is a predicted discrepancy map, whose physical significance lies in locating and highlighting potential enhanced target regions.

Conditional diffusion generation stage: The predicted discrepancy map is concatenated with the original T1-weighted images to form a composite conditional input that contains both anatomical context and pathology-related cues. This condition is then encoded by ControlNet and injected into the reverse denoising process of the diffusion backbone, enabling the model to synthesize high-fidelity T1-CE images under spatially explicit guidance.

Through the above dual constraint mechanism of “first locate, then generate,” DAGCN effectively suppresses the common false enhancement phenomenon in image synthesis while fully utilizing the generative capability of diffusion models through explicit difference guidance.

### 2.2. Difference-Aware Fusion and Prediction Module

**Figure 2:**
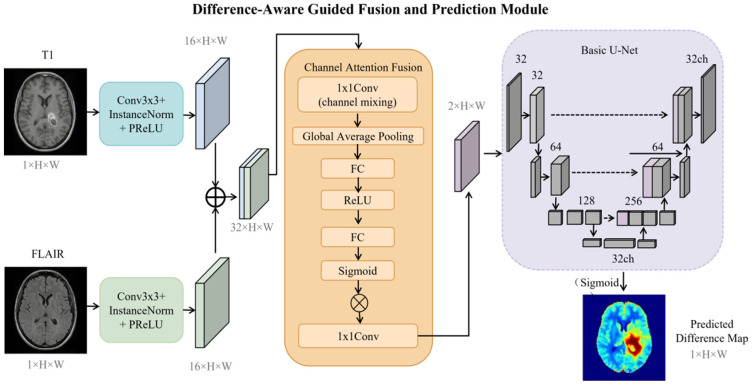
Structure of the Difference-Aware Fusion and Prediction (DAFP) module. T1 and the auxiliary sequence are first processed by two independent convolutional branches for low-level feature extraction. The extracted features are fused through a cross-modal channel attention mechanism and projected into a compact 2-channel bottleneck guidance representation. This representation is then decoded by a Basic U-Net predictor to generate the final single-channel lesion-related discrepancy map used to guide subsequent CE-MRI synthesis.

In the absence of any contrast agent input, the key challenge in contrast-enhanced image synthesis is the accurate localization of potential enhancement regions. To this end, we developed a Difference-Aware Fusion and Prediction (DAFP) module. The module is designed to estimate a lesion-related enhancement increment map by leveraging the complementary information provided by the T1-weighted sequence, which preserves the anatomical background structure, and the auxiliary sequence, which provides complementary lesion-related information. The detailed architecture of the DAFP module is shown in Figure 2.

Dual-Branch Independent Feature Extraction: Given single-channel non-contrast image $I_{T1}$ and auxiliary sequence image $I_{Aux}$(Aux∈{T2,FLAIR}), the module first employs parameter-independent dual-branch convolutional networks for low-level feature extraction. Each branch consists of 3 × 3 convolutional layers, Instance Normalization (IN), and PReLU activation functions. This process can be mathematically expressed as:

(1) $$F_{T1}=\mathrm{PReLU}\left(\mathrm{IN}\left({\mathrm{Conv}}_{3\times 3}\left(I_{T1}\right)\right)\right)\in R^{C\times H\times W}$$

(2) $$F_{Aux}=PReLU\left(\mathrm{IN}\left({\mathrm{Conv}}_{3\times 3}\left(I_{Aux}\right)\right)\right)\in R^{C\times H\times W}$$

where the channel dimension is expanded to C=16. Compared to traditional ReLU, PReLU can effectively avoid the “neuron death” phenomenon caused by negative gradient truncation in deep networks [27]; while Instance Normalization (IN) helps eliminate common contrast shifts in multi-center MRI images at the individual level [28].

To resolve the semantic conflicts easily triggered when features of different physical meanings are directly concatenated, this network introduces a lightweight channel attention mechanism after the spatial concatenation operation. First, the dual-branch features are concatenated in the channel dimension to obtain preliminary fused features ***X***:

(3) $$X=\mathrm{Concat}\left(F_{T1},F_{Aux}\right)\in R^{2C\times H\times W}$$

Subsequently, the spatial dimensions ***X*** are compressed through global adaptive average pooling to extract the global statistical feature $z\in R^{2C}$ vector for each channel. The element for the c-th channel is calculated as follows:

(4) $$z_{c}=\frac{1}{H\times W}\sum_{i=1}^{H}\sum_{j=1}^{W}X_{c,i,j}$$

Then, through a two-layer fully connected network containing dimensionality reduction and dimensionality expansion operations (reduction coefficient r = 4) and Sigmoid activation function, adaptive importance weights for each channel are generated:

(5) $$W_{attn}=\sigma \left(W_{2}\delta \left(W_{1z}\right)\right)$$

where σ is the Sigmoid function, δ is the ReLU function, ***W***_1_ and ***W***_2_ are learnable linear mapping weight matrices. Finally, the attention weights are multiplied with the concatenated features through channel-wise broadcast multiplication. The attended 32-channel representation is then projected by a 1 × 1 convolution into a compact 2-channel bottleneck guidance representation:

(6) $$F_{fused}={\mathrm{Conv}}_{1\times 1}\left(X⊗W_{attn}\right)\in R^{2\times H\times W}$$

This 2-channel representation is not designed to retain all low-level cross-modal texture information. Instead, it provides a compact learnable guidance interface that summarizes enhancement-relevant cues from the attention-recalibrated T1 and auxiliary sequence features while suppressing redundant background responses. The two channels are latent guidance channels and should not be interpreted as manually assigned T1-specific or auxiliary sequence-specific channels.

This attention mechanism dynamically emphasizes informative channel responses in the auxiliary sequence that are closely related to lesion-associated edema, while suppressing redundant anatomical background signals, such as cerebrospinal fluid.

The bottleneck guidance representation is then decoded by the subsequent Basic U-Net predictor. Through multi-scale convolutional encoding and decoding, the predictor expands the compact guidance representation into hierarchical feature maps and finally outputs a single-channel continuous discrepancy map ($P_{diff}$):

(7) $$P_{diff}=\sigma \left(F_{UNet}\left(F_{fused};\theta_{U}\right)\right)\in R^{1\times H\times W}$$

During network training, the ground truth discrepancy map $D_{GT}$ is not constructed by direct subtraction, but introduces a critically important ReLU truncation operator:

(8) $$D_{GT}=\mathrm{ReLU}\left(I_{T1CE}-I_{T1}\right)$$

The ReLU operation was introduced to emphasize positive enhancement-related changes between non-contrast T1 and T1-CE images. This design is based on the assumption that gadolinium uptake generally increases signal intensity in enhancing regions on T1-CE images. Negative differences after preprocessing may arise from residual registration errors, motion artifacts, local noise, or intensity normalization variation, and were therefore not treated as direct enhancement cues in this framework.

However, this non-negative discrepancy target also has limitations. In cases with severe motion, imperfect inter-sequence registration, or poor image quality, a small amount of valid local information may be suppressed by the ReLU operation. Therefore, the ReLU-based discrepancy map should be interpreted as a positive enhancement-prior map rather than a complete representation of all intensity changes between T1 and T1-CE images. Finally, the predicted discrepancy map is constrained to match the ground-truth distribution using an L1 loss:

(9) $$L_{diff}=|P_{diff}-D_{GT}{|}_{1}$$

### 2.3. ControlNet-Guided Diffusion Generation Module

After the enhancement-related target regions have been localized, the second stage of DAGCN uses a ControlNet-guided diffusion model to synthesize the final image. This stage adopts a denoising diffusion probabilistic backbone based on diffusion-model principles [23,29,30] and introduces ControlNet-style spatial conditioning [24], thereby reducing false-positive enhancement during image synthesis.

To ensure that the generated images neither deviate from the real brain anatomical structure nor precisely reflect the predicted pathological enhancement increments, the network constructs a composite conditional input C with channel dimension of 2:

(10) $$C=\mathrm{Concat}\left(I_{T1},\mathrm{Detach}\left(P_{diff}\right)\right)\in R^{2\times H\times W}$$

During training, the predicted discrepancy map was detached before being used as the ControlNet condition. This operation prevents the gradients from the diffusion-generation loss from being backpropagated into the discrepancy prediction branch. The motivation for this design was to stabilize the two-stage optimization and preserve the interpretability of the discrepancy predictor, whose target is defined as a positive enhancement-related difference map.

However, this design also introduces a trade-off. Because of the Detach() operation, the discrepancy predictor is mainly supervised by the discrepancy-map reconstruction loss and does not directly receive feedback from the final image generation quality. Therefore, although gradient detachment improves training stability and helps avoid feature drift in the discrepancy prediction branch, it may limit the potential benefit of fully coupled optimization.

ControlNet Spatial Prior Injection: Traditional diffusion models often exhibit strong randomness when executing reverse denoising. To achieve deterministic conditional constraints, this model introduces the ControlNet architecture. ControlNet replicates the encoder structure and weights of the backbone Diffusion U-Net, specifically designed to receive and encode the aforementioned dual-channel composite conditions C. Through the front-end zero convolution feature embedding layer, condition information is transformed into multi-scale spatial prior features and injected layer by layer into the decoder of the Diffusion U-Net. This spatial conditioning encourages the diffusion model to synthesize enhancement signals only within the “highlighted” high-weight regions activated by $P_{diff}$, while strictly following the T1 baseline gray distribution in non-contrast regions.

Diffusion Denoising and Multi-Task Joint Optimization: During reverse denoising sampling, following the iterative denoising principle of diffusion-based generation and restoration frameworks [23,29], the diffusion network starts from a pure Gaussian noise distribution $I_{T}^{\left(noise\right)}$ and, within the given time step t (set to T = 1000 steps in this study), it progressively removes the prediction noise $\epsilon_{\theta }$ under the guidance of condition C, finally recovering realistic $I_{T1CE}$ images. The generation loss $L_{gen}$ during model training uses Mean Squared Error (MSE) to measure the deviation between the network-predicted noise and the actual added noise ϵ during forward diffusion:

(11) $$L_{gen}=|\epsilon -\epsilon_{\theta }\left(I_{t},t,C\right){|}_{2}^{2}$$

To balance the optimization dynamics of the two-stage framework, we use a weighted joint loss for cooperative multi-task optimization. This decoupled yet cooperative design makes it possible to exploit modality-specific prior information while maintaining both local lesion fidelity and global anatomical plausibility in the synthesized images:

(12) $$L_{total}=\lambda_{gen}L_{gen}+\lambda_{diff}L_{diff}$$

The loss weights were set to $\lambda_{gen}=10$ and $\lambda_{diff}$ = 1. These values were not obtained through an exhaustive grid search or randomized hyperparameter search. Instead, they were selected through limited empirical tuning based on common practice in balancing the main diffusion-generation loss and the auxiliary discrepancy prediction loss, together with monitoring of training stability, validation-loss convergence, and visual plausibility of the predicted discrepancy maps. Specifically, $\lambda_{gen}$ was assigned a larger weight to emphasize the fidelity of the denoising-based image generation task, whereas $\lambda_{diff}$ was kept at 1 to provide direct but balanced supervision for discrepancy-map prediction. Under this setting, the model showed stable convergence and produced visually interpretable discrepancy maps. A controlled sensitivity analysis over alternative loss-weight combinations was not performed because of the high computational cost of diffusion-model training.

### 2.4. Dataset Construction, Slice Selection, and Data Splitting

This study was conducted using the Brain Tumor Segmentation Challenge (BraTS 2021) dataset, a widely recognized benchmark in medical image analysis [31,32,33]. For each case, four co-registered MRI modalities were available, including non-contrast T1-weighted imaging (T1), T2-weighted imaging (T2), fluid-attenuated inversion recovery (FLAIR), and gadolinium-enhanced T1-weighted imaging (T1-CE). The T1-CE image was used as the target reference for supervised synthesis. Although BraTS 2021 was not designed specifically as an image-synthesis challenge, it provides paired multiparametric brain MRI suitable for supervised synthesis, while public synthesis benchmarks such as SynthRAD2023 further emphasize the importance of standardized datasets and transparent evaluation in medical image synthesis [34].

All data splitting was performed strictly at the patient level to avoid data leakage. Paired T1, T2, FLAIR, and T1-CE images from the same subject were always assigned to the same subset. The official BraTS 2021 training cohort was used for model development and was divided into a training subset and an internal validation subset at an 8:2 ratio. Specifically, 1000 patients were used for model training, and 251 patients were used for internal validation. For each patient in the training and internal validation subsets, the middle 50 axial slices were selected to construct paired training or validation samples. The internal validation subset was used for validation-loss monitoring and checkpoint selection.

The official BraTS 2021 validation cohort was not used for model training, hyperparameter tuning, or checkpoint selection. It was kept separate and used as an independent held-out test source. To reduce the computational burden of evaluating all baseline methods while maintaining a consistent comparison, 50 patients were selected from this held-out test source. The 50 cases were selected deterministically by sorting the 219 validation cases according to anonymized BraTS case identifiers and taking the first 50 cases; no manual selection based on tumor appearance, enhancement degree, image quality, model output, or quantitative performance was applied. For each selected patient, the middle 50 axial slices were used, resulting in 2500 paired test slices for each input setting. For paired-sample construction, the non-contrast T1 image, auxiliary sequence image (T2 or FLAIR), and corresponding T1-CE image were organized as input-target triplets. The overall dataset construction, patient-level allocation, slice-selection strategy, and sample numbers are summarized in Table 1.

Data preprocessing was implemented using MONAI. First, intensity clipping was applied using the 0.5th and 99.5th percentiles to reduce the influence of extreme outliers. All images were then linearly normalized to the range [0, 1] and resized to 240 × 240 pixels. The same preprocessing pipeline was applied to the training, internal validation, and held-out test data.

### 2.5. Experimental Environment and Parameter Selection

Training and inference were conducted on a deep learning workstation equipped with eight NVIDIA RTX 3090 GPUs (24 GB memory each). The core implementation was based on the PyTorch deep learning framework, with integration of MONAI (Medical Open Network for AI) and its Generative Models extension library for medical image analysis [35]. Training and inference were implemented in PyTorch (version 1.13.0+cu116), MONAI (version 1.3.2), and MONAI Generative Models (version 0.2.3).

During optimization, the Adam optimizer was used to jointly update all learnable parameters in the discrepancy prediction module, the cross-modal channel-attention fusion module, and the two diffusion networks, namely the Diffusion U-Net and ControlNet. The initial learning rate was set to 1 × 10^−4^. A batch size of 4 was selected because it was the largest stable batch size that could be accommodated by the 24 GB RTX 3090 GPUs while maintaining stable diffusion-model training on 240 × 240 slices.

**Figure 3:**
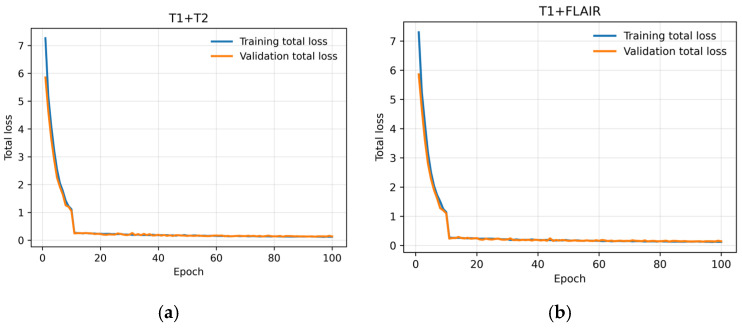
Training and validation total loss curves under the two auxiliary sequence settings. (**a**) Loss curves under the T1 + T2 setting. (**b**) Loss curves under the T1 + FLAIR setting. In both settings, the training and validation total losses decreased rapidly during the early training stage and then gradually converged. The minimum validation total loss was obtained at epoch 94 for both T1 + T2 and T1 + FLAIR, and the corresponding checkpoints were selected for final testing.

The model was trained for a maximum of 100 epochs. During training, both training total loss and validation total loss were monitored, and the final testing checkpoint was selected according to the lowest validation total loss rather than the last epoch alone. As shown in Figure 3, the training and validation losses decreased rapidly during the early training stage and then gradually converged under both the T1 + T2 and T1 + FLAIR settings. The minimum validation total loss was 0.129579 at epoch 94 for the T1 + T2 setting and 0.132047 at epoch 94 for the T1 + FLAIR setting, indicating stable optimization without evident overfitting.

For the core hyperparameter configuration of the conditional diffusion generation process, the total number of diffusion timesteps for forward noise addition and reverse denoising was fixed at T = 1000, following the standard DDPM configuration. This setting was kept identical for the DDPM baseline and DAGCN-based models to ensure a consistent diffusion-sampling protocol across diffusion-based comparisons. A dedicated timestep-ablation or reduced-step sampler analysis was not performed in the current study. For the weighted multi-task loss, the diffusion-generation loss weight was set to $\lambda_{gen}=10$, and the discrepancy prediction loss weight was set to $\lambda_{\mathrm{diff}}=1$, consistent with the description in Section 2.3. These values were selected through limited empirical tuning rather than exhaustive grid or randomized search. During preliminary training, we monitored training stability, validation-loss convergence, and the visual plausibility of the predicted discrepancy maps and synthesized T1-CE images. A full sensitivity analysis over different loss-weight combinations was not performed because of the high computational cost of diffusion-model training.

### 2.6. Baseline Models and Modern Synthesis Comparisons

To provide a comprehensive comparison with both image-fusion and medical image synthesis approaches, we evaluated DAGCN against six baseline methods. The image-fusion baselines included U2Fusion, DIDFuse, and SwinFusion, which were retained because they represent commonly used multi-modal fusion strategies for combining non-contrast T1 with T2 or FLAIR. In addition, three modern synthesis baselines, including cGAN, Hi-Net, and conditional DDPM, were added to address comparison with CE-MRI-oriented or generative medical image synthesis approaches.

All baseline methods were trained and evaluated under the same T1 + T2 and T1 + FLAIR input settings using the same preprocessing pipeline, data split, test samples, and quantitative metrics. For the conditional DDPM baseline, the same diffusion backbone was used without the proposed Difference-Aware Fusion and Prediction module or ControlNet-guided spatial conditioning. This design allowed the contribution of discrepancy-aware localization and ControlNet-guided generation to be assessed more directly.

### 2.7. Statistical Analysis

Statistical analysis was performed at the patient level to avoid treating multiple slices from the same patient as independent observations. For each selected patient, each metric was first averaged across the middle 50 axial slices. Paired Wilcoxon signed-rank tests were then performed between DAGCN and each baseline method using the per-patient mean metrics (*n* = 50 patients) for PSNR, SSIM, VIF, and NCC under both T1 + T2 and T1 + FLAIR settings. Holm–Bonferroni correction was applied for multiple comparisons within each input setting and metric. An adjusted *p*-value < 0.05 was considered statistically significant.

### 2.8. Computational Efficiency Profiling

To evaluate the practical feasibility of the proposed framework, we measured the computational efficiency of DAGCN, the baseline methods, and the DAGCN ablation variants. The evaluated indicators included trainable parameter count, multiply–accumulate operations (MACs), forward inference time or single-step denoising time, and peak GPU memory usage.

All measurements were performed under the T1 + FLAIR setting using a single NVIDIA RTX 3090 GPU, with an input image size of 240 × 240 and a batch size of 1. Because the T1 + T2 and T1 + FLAIR settings have the same input tensor size and channel number, this profiling reflects the computational cost of the two-input setting. For each model, 10 warm-up runs and 50 repeated measurements were performed, and the average runtime was reported. Each model was profiled in an isolated build-measure-delete cycle with CUDA cache cleanup. For cGAN, Hi-Net, U2Fusion, DIDFuse, and SwinFusion, runtime refers to the forward inference time. For the vanilla conditional diffusion baseline and DAGCN-based models, runtime refers to single-step denoising time because complete sampling requires iterative denoising.

## 3. Results

### 3.1. Experimental Results Comparison and Analysis

The image synthesis performance of the proposed DAGCN model was systematically evaluated on the independent BraTS 2021 test set. Two input configurations, namely T1 + T2 and T1 + FLAIR, were investigated. To address both medical image synthesis and multi-modal fusion perspectives, the proposed method was compared with cGAN [36], Hi-Net [37], conditional DDPM [30], U2Fusion [38], DIDFuse [39], and SwinFusion [40]. All methods were evaluated under the same preprocessing pipeline, test samples, and quantitative metrics. The full method set was evaluated using objective quantitative, patient-level statistical, computational-efficiency, and main visual-comparison analyses, while radiologist-based subjective evaluation was performed on three representative and comparatively competitive baseline methods and ablation variants to keep the reader study feasible. The quantitative metrics included PSNR, SSIM, VIF, and NCC, while radiologist-based subjective evaluation included image quality (IQ), clinical replaceability (IC), contrast enhancement (CE), and lesion conformity (CF). For subjective evaluation, the synthesized images were anonymized and randomly ordered before review. The radiologists were informed that the study involved image quality evaluation of synthesized contrast-enhanced MRI, but they were blinded to the generation method, input setting, and model variant. No method labels were provided during scoring.

#### 3.1.1. Qualitative Visual Comparison of Synthesis Methods

**Figure 4:**
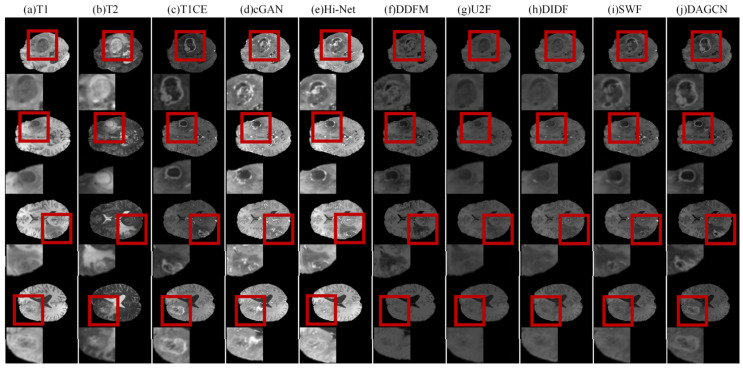
Visual comparison of synthesized T1-CE images under the T1 + T2 input setting. The figure includes synthesis-specific baselines (cGAN, Hi-Net, and DDPM), image-fusion baselines (U2Fusion, DIDFuse, and SwinFusion), and the proposed DAGCN. Compared with baseline methods, DAGCN better preserves lesion-localized enhancement patterns while reducing false-positive enhancement and maintaining global anatomical consistency.

**Figure 5:**
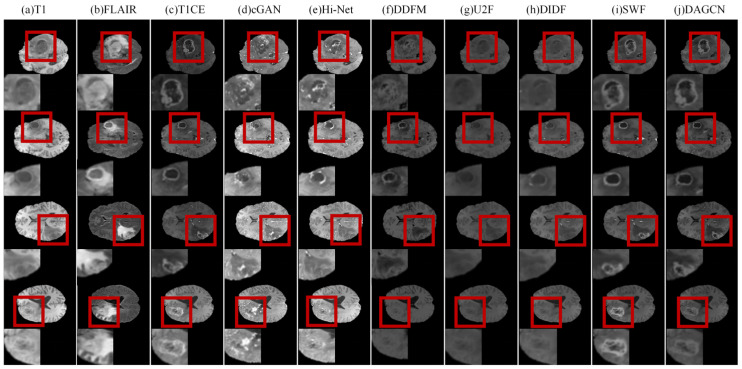
Visual comparison of synthesized T1-CE images under the T1 + FLAIR input setting. Results from cGAN, Hi-Net, DDPM, U2Fusion, DIDFuse, SwinFusion, and DAGCN are shown together with the real T1-CE reference. DAGCN produces cleaner background regions and more spatially focused lesion enhancement, particularly benefiting from the CSF-suppression property of FLAIR guidance.

Figure 4 and Figure 5 present representative synthesis results under the T1 + T2 and T1 + FLAIR settings, respectively. U2Fusion produced overly smooth images with insufficient lesion enhancement, making subtle abnormalities difficult to detect. DIDFuse recovered more high-frequency detail but introduced intensity discontinuities and boundary artifacts. SwinFusion preserved global anatomy relatively well, yet enhancement in deep brain regions remained incomplete. In contrast, DAGCN generated images with a more convincing balance between global anatomical structure and local lesion conspicuity.

**Figure 6:**
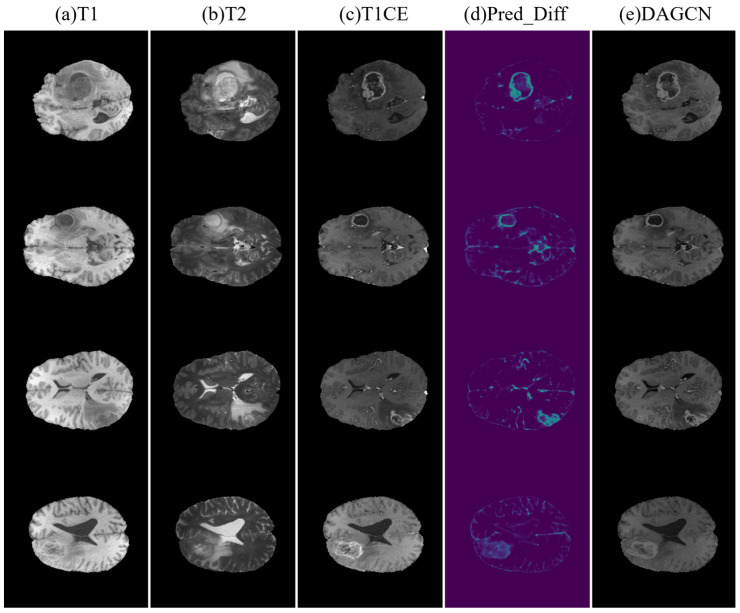
Representative generation process of DAGCN under T2-guided synthesis. For each case, the non-contrast T1-weighted images, auxiliary T2 image, predicted discrepancy map, synthesized T1-CE image, and ground-truth T1-CE image are shown for comparison. Although the predicted discrepancy maps generally localize the lesion region, residual interference from cerebrospinal fluid and nonspecific hyperintense areas may still be observed under T2 guidance. Predicted discrepancy maps are displayed using the perceptually uniform viridis sequential colormap with a consistent scale, following recommendations for scientific color use [42].

**Figure 7:**
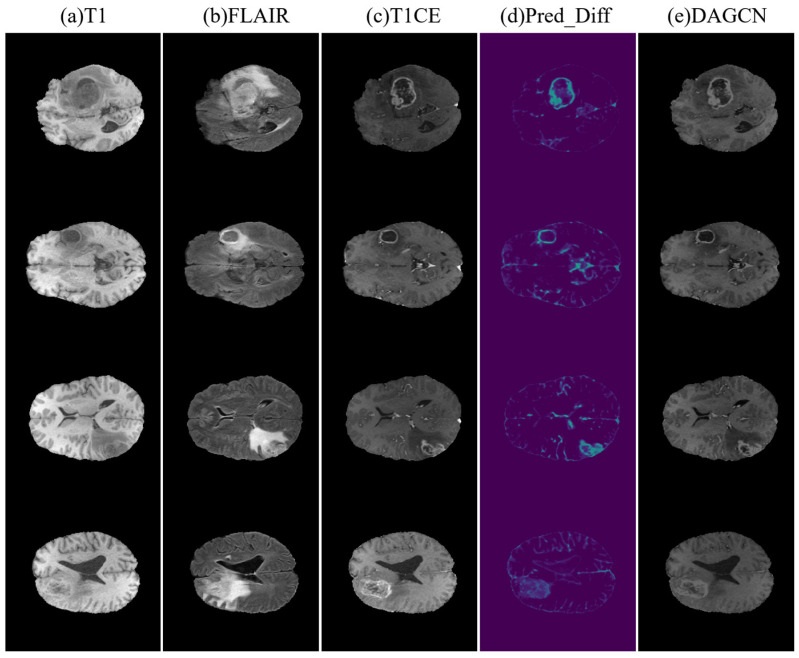
Representative generation process of DAGCN under FLAIR-guided synthesis. For each case, the non-contrast T1-weighted images, auxiliary FLAIR image, predicted discrepancy map, synthesized T1-CE image, and ground-truth T1-CE image are shown. Compared with T2 guidance, FLAIR guidance yields more spatially focused discrepancy maps, cleaner background suppression, and better delineation of enhancing lesion boundaries. Predicted discrepancy maps are displayed using the perceptually uniform viridis sequential colormap with a consistent scale, following recommendations for scientific color use [42].

To further investigate the differences in DAGCN under the two auxiliary modalities, Figure 6 and Figure 7 illustrate the detailed synthesis process for four representative cases under T2-guided and FLAIR-guided settings, respectively. As shown in Figure 6, under T2 guidance, the predicted discrepancy maps (fourth column) successfully identified the main enhancing regions of the tumors in all four cases. Nevertheless, because cerebrospinal fluid and nonspecific white matter hyperintensities also exhibit high signal intensity on T2-weighted images [41], the discrepancy prediction network was unable to completely separate pathological enhancement from normal free-water-related signals.

As shown in Figure 7, under the FLAIR-guided setting, the synthesis process of the same four representative cases displayed distinctly different characteristics. The most apparent difference between the FLAIR sequence (second column) and the T2 sequence was observed in the ventricular region: cerebrospinal fluid, which appears bright on T2-weighted images, was effectively suppressed on FLAIR, while the peritumoral vasogenic edema remained hyperintense. This physical imaging characteristic was directly reflected in the predicted discrepancy maps. A comparison of the Pred_Diff columns for the same cases in Figure 6 and Figure 7 shows that the discrepancy maps under FLAIR guidance were more spatially concentrated and better localized.

These findings suggest that the choice of auxiliary sequence has a direct impact on the output quality of the discrepancy prediction module and, in turn, on the final image synthesis performance. The T2-weighted sequence provides rich information on tissue water content, which is useful for identifying the approximate extent of the lesion; however, its nonspecific sensitivity to free-water signals may lead to redundant activations in the discrepancy map. By contrast, the FLAIR sequence suppresses cerebrospinal fluid signals, allowing the discrepancy prediction network to focus more selectively on pathological tissue changes and thereby produce discrepancy maps with higher specificity. As a result, the diffusion model guided by these maps yields synthesized images with more accurate lesion boundaries and cleaner background appearance.

#### 3.1.2. Quantitative and Statistical Analysis of Synthesis Methods

**Figure 8:**
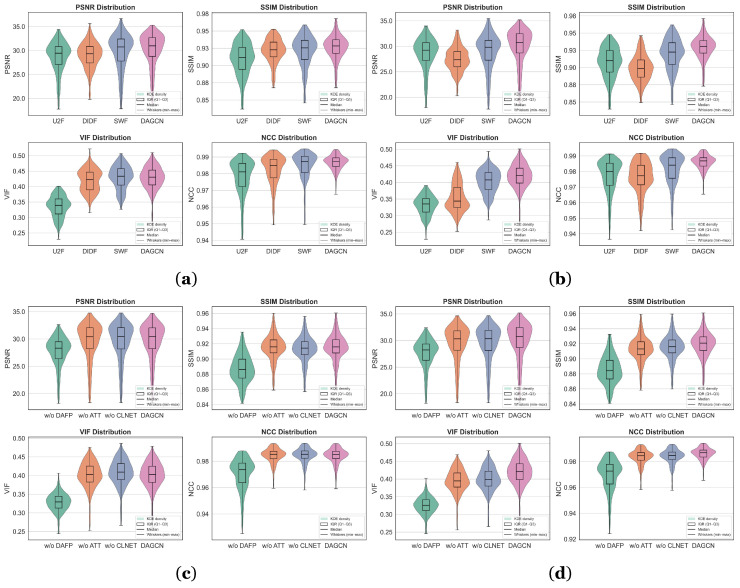
Quantitative performance distributions of representative and comparatively competitive baseline methods and ablation variants. Violin plots show the distributions of PSNR, SSIM, VIF, and NCC values. (**a**) Comparison of representative methods under the T1 + T2 setting. (**b**) Comparison of representative methods under the T1 + FLAIR setting. (**c**) Ablation results under the T1 + T2 setting. (**d**) Ablation results under the T1 + FLAIR setting.

Table 2 summarizes the quantitative performance of all evaluated methods, including cGAN, Hi-Net, DDPM, U2Fusion, DIDFuse, SwinFusion, and DAGCN, in terms of PSNR, SSIM, VIF, and NCC under the T1 + T2 and T1 + FLAIR input settings. To improve readability, the quantitative ablation results are reported separately in Table 3. Considering the large number of compared methods and to preserve figure readability and reader-study feasibility, the violin plot distributions in Figure 8a,b and the radiologist-based subjective evaluation were retained as representative analyses of three comparatively competitive baseline methods (U2Fusion, DIDFuse, and SwinFusion) and DAGCN; the full baseline set, including cGAN, Hi-Net, and DDPM, is reported in Table 2 and Table 4.

As shown in Table 2, DAGCN achieved the highest PSNR and NCC under both input settings. Under the T1 + T2 setting, DAGCN achieved a PSNR of 30.011 ± 3.605 dB and an NCC of 0.986 ± 0.004. Under the T1 + FLAIR setting, DAGCN achieved a PSNR of 29.869 ± 3.540 dB and an NCC of 0.985 ± 0.004. The expanded visual comparisons in Figure 4 and Figure 5 include cGAN, Hi-Net, DDPM, U2Fusion, DIDFuse, SwinFusion, and DAGCN. These comparisons indicate that DAGCN provides more accurate lesion-localized enhancement and cleaner background suppression than the baseline methods, while maintaining global anatomical consistency. Although SSIM and VIF were not uniformly superior across all baselines, DAGCN showed competitive performance in these metrics, suggesting a balanced improvement in intensity fidelity, structural consistency, and lesion-focused enhancement synthesis.

To further assess whether the observed quantitative differences remained statistically significant after accounting for within-patient correlation, each metric was first averaged within each patient and paired Wilcoxon signed-rank tests were then performed at the patient level (*n* = 50). The patient-level *p*-values are summarized in Table 4.

As shown in Table 2, DAGCN achieved significantly higher PSNR and NCC than all compared baseline methods under both T1 + T2 and T1 + FLAIR settings. For SSIM and VIF, DAGCN showed competitive performance but not uniform superiority over all baselines. Specifically, no significant difference was observed between DAGCN and DIDFuse in SSIM under the T1 + T2 setting, or between DAGCN and SwinFusion in SSIM under the T1 + FLAIR setting. Therefore, the quantitative findings were interpreted as evidence of improved intensity fidelity and structural consistency, rather than uniform superiority across all image quality metrics.

Under the T1 + T2 setting, DAGCN achieved the highest PSNR of 30.011 ± 3.605 dB and the highest NCC of 0.986 ± 0.004, indicating improved intensity fidelity and strong structural consistency. However, DAGCN did not achieve uniform superiority across all metrics. Its SSIM value of 0.921 ± 0.017 was comparable to that of DIDFuse and lower than that of SwinFusion. In addition, although DAGCN showed competitive VIF performance among the image-fusion baselines, some newly added medical image synthesis baselines showed higher VIF values. Therefore, the quantitative advantage of DAGCN should be interpreted mainly in terms of PSNR and NCC, together with lesion-focused visual quality and radiologist-based evaluation, rather than as across-the-board superiority in every whole-image metric.

Under the T1 + FLAIR setting, DAGCN achieved the highest PSNR of 29.869 ± 3.540 dB and the highest NCC of 0.985 ± 0.004 among all compared methods. Its SSIM was comparable to that of SwinFusion, while its VIF remained competitive but was not uniformly superior to all baselines. These findings indicate that DAGCN provides stable intensity fidelity and structural consistency under FLAIR guidance. Meanwhile, the visual comparison and representative radiologist-based subjective evaluation further suggest that the main benefit of FLAIR-guided DAGCN lies in more focused lesion localization and cleaner background suppression.

From a clinical standpoint, the T1 + T2 and T1 + FLAIR settings showed different but complementary characteristics. The T1 + T2 setting achieved slightly higher absolute values for some whole-image quantitative metrics, suggesting favorable global pixel-level fidelity. However, the visual analysis indicated that T2 guidance was more susceptible to redundant activations related to cerebrospinal fluid and nonspecific free-water signals, which could occasionally lead to periventricular false-positive enhancement. In contrast, FLAIR guidance provided stronger suppression of cerebrospinal fluid signals and produced more spatially focused discrepancy maps, resulting in clearer lesion boundaries and cleaner background appearance. These findings suggest that T2 guidance may provide broader edema-related anatomical cues, whereas FLAIR guidance may offer higher specificity for lesion-related enhancement. Therefore, the two auxiliary sequences provide distinct guidance information for CE-MRI synthesis, and their effects should be interpreted in relation to both global quantitative metrics and local lesion-specific image quality.

To further evaluate computational feasibility, we compared the model complexity and inference efficiency of DAGCN with the baseline methods and ablation variants. The evaluated indicators included trainable parameter count, MACs, forward inference time or single-step denoising time, and peak GPU memory usage. The results are summarized in Table 5.

As shown in Table 5, DAGCN required 92.10 M trainable parameters, 212.96 G MACs, 47.05 ms per denoising step, and 1193.87 MB peak inference GPU memory. Compared with lightweight CNN-based or fusion-based baselines, DAGCN introduced additional computational overhead because it integrates a diffusion backbone with ControlNet-based spatial conditioning. Compared with the w/o CLNET variant, the full DAGCN increased the parameter count from 65.13 M to 92.10 M and the MACs from 165.15 G to 212.96 G. By contrast, the full DAGCN and the w/o ATT variant showed nearly identical parameter counts, MACs, denoising time, and peak inference memory because the w/o ATT variant only replaces the lightweight cross-modal attention-fusion block with a simple concatenation-based fusion block, while the downstream discrepancy predictor, ControlNet, and diffusion U-Net remain unchanged. Therefore, the w/o ATT ablation should be interpreted primarily as a test of feature-fusion quality rather than computational efficiency. The additional computational cost of the full DAGCN was mainly associated with ControlNet-based spatial conditioning and diffusion generation, whereas the benefit of attention fusion was reflected in lesion localization and synthesis fidelity, as supported by the ablation results.

Because diffusion-based models require iterative denoising, the effective sampling time depends on the number of inference steps. Under the current 1000-step sampling setting, the estimated sampling time was approximately 47.05 s per slice for DAGCN. Therefore, the current implementation is more suitable for offline or semi-automated CE-MRI synthesis workflows rather than real-time clinical deployment. Future work will investigate accelerated sampling strategies, including DDIM sampling, reduced denoising steps, and latent diffusion [43], to improve clinical efficiency.

#### 3.1.3. Radiologist-Based Evaluation of Synthesis Methods

Two neuroradiologists with 8 and 10 years of subspecialty experience independently evaluated the synthesized images. Before evaluation, all images were anonymized and randomly ordered. The readers were informed that the study involved image quality evaluation of synthesized contrast-enhanced MRI, but they were blinded to the generation method, input setting, and model variant. No method labels were provided during scoring.

A 5-point Likert scale was used to evaluate four criteria: image quality (IQ), clinical replaceability (IC), contrast enhancement (CE), and lesion conformity (CF). The scoring scale was defined as follows: 1, non-diagnostic or clearly unacceptable synthesis; 2, poor image quality with major artifacts or unreliable lesion depiction; 3, acceptable but visibly imperfect synthesis; 4, good image quality with minor defects; and 5, image quality and lesion depiction close to real contrast-enhanced T1-weighted MRI.

The radiologist-based subjective scores are summarized in Table 6. The table reports the mean ± standard deviation values for image quality (IQ), clinical replaceability (IC), contrast enhancement (CE), and lesion conformity (CF) under representative algorithm-comparison and ablation-analysis settings; the algorithm-comparison reader study included three comparatively competitive baseline methods and DAGCN.

Inter-rater reliability between the two neuroradiologists was evaluated using ICC(2,1), a two-way random-effects absolute-agreement single-measure intraclass correlation coefficient. The overall ICC across all subjective ratings was 0.895 (95% CI: 0.890–0.901), indicating good inter-rater reliability. Criterion-specific ICC values were 0.811 for IQ, 0.901 for IC, 0.930 for CE, and 0.892 for CF, indicating good-to-excellent reliability across the four scoring dimensions. Nevertheless, the two-reader design remains a limitation and should be expanded in future multi-reader, multi-center validation studies. The full ICC results are summarized in Table 7.

As shown in Table 6, DAGCN achieved higher subjective scores than the representative and comparatively competitive baseline methods in most evaluation domains under both T1 + T2 and T1 + FLAIR settings. In particular, the T1 + FLAIR configuration showed higher IC and CF scores than the T1 + T2 configuration, indicating improved perceived clinical usability and lesion conformity. In the ablation analysis, removal of the DAFP module resulted in the lowest CE scores, suggesting that explicit discrepancy prediction is important for localizing enhancement-related regions. These subjective results were consistent with the representative visual comparisons and quantitative findings.

Under the T1 + T2 setting, DAGCN achieved the highest scores across all four evaluation criteria compared with the other representative baseline methods included in the reader study (Table 6). Nevertheless, the radiologists noted that a few cases still exhibited slight over-enhancement in the periventricular region, which is consistent with the cerebrospinal fluid-related interference observed in the preceding visual analysis of the T2-guided setting.

Under the T1 + FLAIR setting, DAGCN achieved the highest IQ, IC, and CF scores among the representative methods included in the reader study, while SwinFusion showed a slightly higher CE score (Table 6). The radiologists particularly emphasized two observations: first, the boundary between the enhancing tumor region and the surrounding edema was delineated more clearly, without obvious overextension of enhancement into normal tissue; second, the periventricular over-enhancement observed in some T1 + T2 cases was completely eliminated under FLAIR guidance, making the synthesized images diagnostically closer to the real T1-CE images.

Comparing the two input settings for DAGCN, all four subjective scores were higher under T1 + FLAIR than under T1 + T2. This finding complements the objective quantitative results, in which the T1 + T2 setting showed slightly better whole-image statistics. The higher purity and stronger spatial focus of the discrepancy maps under FLAIR guidance allowed the synthesized images to perform better in local lesion regions, which are of greatest clinical relevance, and this likely explains the consistently higher clinical scores. Notably, among the representative and comparatively competitive baseline methods included in the reader study, SwinFusion also showed a markedly higher CE score under T1 + FLAIR (4.82) than under T1 + T2 (4.19), further suggesting that FLAIR provides a general guidance benefit for image synthesis. By incorporating discrepancy-aware guidance, DAGCN was able to translate this benefit more effectively into improved clinical image quality.

Taken together, the quantitative results, visual analysis, and representative radiologist-based subjective evaluation consistently indicate that FLAIR offers clear advantages in lesion localization specificity, background cleanliness, and diagnostic quality, and can therefore be considered the preferred auxiliary sequence for T1-CE image synthesis.

#### 3.1.4. Failure-Case Analysis

**Figure 9:**
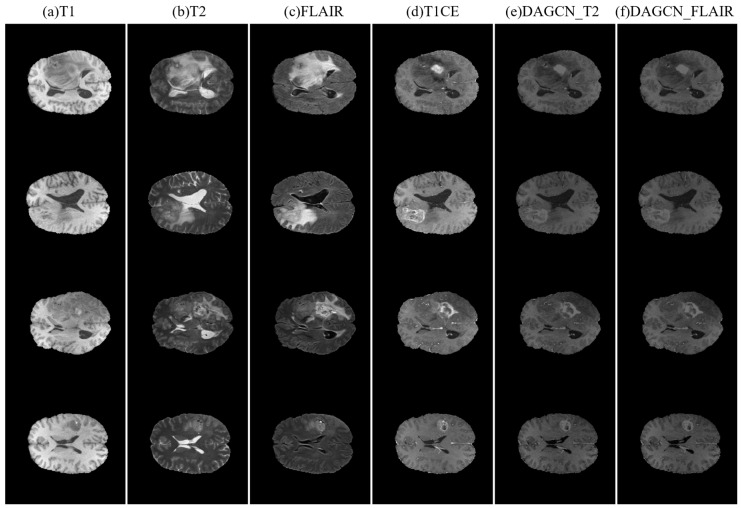
Representative failure cases. From left to right, each row shows the non-contrast T1 image, auxiliary T2 image, auxiliary FLAIR image, acquired T1-CE reference, and DAGCN-generated T1-CE outputs under the two auxiliary sequence settings. The four examples illustrate small focal enhancement, irregular peripheral/rim-like enhancement, heterogeneous enhancement, and small ring- or nodular-enhancing patterns. DAGCN generally preserved the approximate lesion location but underestimated enhancement intensity, smoothed lesion boundaries, and partially reduced internal enhancement heterogeneity.

To further characterize potential failure modes, representative challenging cases were reviewed in Figure 9. The four rows illustrate different patterns in which the synthesized T1-CE images remained anatomically plausible but did not fully reproduce the reference enhancement. In the first row, a small focal enhancing region adjacent to a larger T2/FLAIR abnormality was detected at the correct approximate location, but the synthesized enhancement was visibly weaker and spatially smoother than the real T1-CE image. In the second row, the real T1-CE image shows an irregular peripheral/rim-like enhancing lesion in the posterior region; DAGCN reproduced the lesion region but attenuated the rim intensity and blurred the internal low-signal component, thereby reducing enhancement heterogeneity. In the third row, the reference image contains a more heterogeneous and irregular enhancement pattern; the synthesized result preserved the main enhancing area but partially lost small high-intensity foci and produced a smoother lesion boundary. In the fourth row, a small ring- or nodular-enhancing component was generated at the expected site, but the rim was less sharp and the lesion extent was slightly contracted compared with the acquired T1-CE image. These examples indicate that DAGCN is more likely to fail by underestimating subtle enhancement, smoothing irregular margins, or suppressing internal heterogeneity rather than by producing gross anatomical distortion. Therefore, synthetic T1-CE images should be interpreted cautiously when subtle enhancement, rim morphology, or internal enhancement heterogeneity is clinically decisive.

### 3.2. Ablation Experimental Results Comparison and Analysis

To validate the effectiveness of each core component in the DAGCN framework, three ablation variants were designed under the same hyperparameter settings and evaluated under both T1 + T2 and T1 + FLAIR input configurations. These variants were intended to assess the functional contribution of each component to enhancement localization and image synthesis quality, rather than to serve as separate lightweight efficiency models. The three variants were defined as follows:

Without the Difference-Aware Fusion and Prediction module (w/o DAFP): the entire discrepancy prediction branch was removed, reducing the network to a diffusion-based generation model conditioned only on the non-contrast T1-weighted images as a single-channel input, without using any auxiliary sequence information.

Without cross-modal attention fusion (w/o ATT): the discrepancy prediction branch was retained, but the channel-attention fusion mechanism in the DAFP module was replaced with simple channel concatenation followed by a 1 × 1 convolution. The downstream Basic U-Net discrepancy predictor, ControlNet branch, and diffusion U-Net were kept unchanged. Therefore, this variant was designed to evaluate whether attention-based cross-modal feature recalibration improves discrepancy-map quality, rather than to substantially reduce computational complexity.

Without ControlNet guidance (w/o CLNET): the independent ControlNet encoding branch was removed, and the T1-weighted images were directly concatenated with the predicted discrepancy map before being fed into the backbone Diffusion U-Net for denoising.

#### 3.2.1. Visual Assessment of Ablation Variants

**Figure 10:**
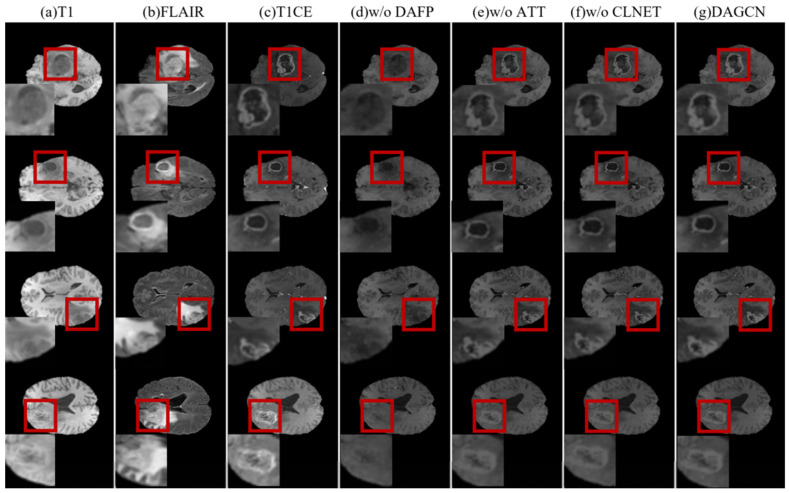
Visual comparison of ablation variants of DAGCN. Representative synthesized T1-CE images are shown for the full model and three ablation settings: without the Difference-Aware Fusion and Prediction module (w/o DAFP), without cross-modal attention fusion (w/o ATT), and without ControlNet guidance (w/o CLNET). Red boxes indicate representative lesion regions where removal of DAFP, cross-modal attention fusion, or ControlNet guidance leads to missed enhancement, boundary overestimation, or degraded intralesional texture.

Figure 10 directly illustrates the impact of removing each component on the final synthesized images (Gen_T1CE). The w/o DAFP variant produced the poorest image quality. Because lesion-localization information provided by the auxiliary sequence was completely lost, this variant generated images that remained close to the non-contrast state in some cases, with little or no effective enhancement in the tumor region. In other cases, irregular hyperintense artifacts were observed, particularly at the gray-white matter junction. These two failure patterns correspond to clinically meaningful missed enhancement and false-positive enhancement, respectively, both of which would substantially compromise diagnostic interpretation.

The w/o ATT variant retained discrepancy prediction capability, and enhancement signals could still be observed in the lesion region. However, because simple concatenation could not effectively suppress redundant information from the auxiliary sequence, activation regions extending beyond the true enhancement range appeared in the predicted discrepancy maps. As a result, the synthesized T1-CE images showed overextended lesion boundaries into surrounding tissues, especially in the periventricular and white matter regions.

The w/o CLNET variant largely preserved the global anatomical structure, but the reconstruction quality in local lesion regions was reduced. In particular, some small enhancing nodules exhibited irregular boundaries, and the grayscale distribution within the enhancement region became less homogeneous. This degradation is likely related to the increased randomness of the denoising process when spatial constraints from ControlNet are absent.

#### 3.2.2. Quantitative Analysis of Ablation Variants

As shown in the figure, all ablation variants showed performance degradation relative to the full DAGCN, but the magnitude and interpretation of the degradation differed across components. The w/o DAFP variant produced the largest decline because it removed explicit discrepancy-map prediction and largely eliminated enhancement target localization. The w/o ATT and w/o CLNET variants showed more moderate but consistent degradation, indicating that cross-modal feature recalibration and ControlNet-based spatial conditioning refine, rather than replace, the basic discrepancy prediction pathway. The decline was more pronounced under the T1 + FLAIR setting, suggesting that the larger modality gap between FLAIR and T1 places greater demands on coordinated interaction among the network components. Although the FLAIR sequence provides more informative lesion priors, these advantages can only be fully exploited when supported by the complete network architecture.

#### 3.2.3. Radiologist-Based Evaluation of Ablation Variants

In addition, two senior neuroradiologists independently performed blinded evaluations of the images generated by each ablation variant. The results under the T1 + T2 and T1 + FLAIR settings are summarized in the ablation rows of Table 6.

Under the T1 + T2 setting, the w/o DAFP variant failed to generate recognizable enhancement signals within the tumor region and therefore had little diagnostic value. Although the w/o ATT variant produced visible enhancement in the lesion area, the estimated boundaries often extended beyond the true pathological extent. The w/o CLNET variant showed overall performance similar to that of w/o ATT, but was mainly characterized by degraded intralesional texture and irregular boundaries of small enhancing nodules.

Under the T1 + FLAIR setting, the w/o DAFP variant showed almost no noticeable change after switching the auxiliary sequence, indicating that the network had largely lost its ability to utilize auxiliary sequence information in the absence of the discrepancy prediction module. By contrast, the w/o ATT variant achieved higher scores across multiple evaluation criteria than under the T1 + T2 setting, suggesting that the intrinsic lesion sensitivity of the FLAIR sequence could partially compensate for the information loss caused by removal of the attention mechanism. Taken together, the ablation results support three main conclusions. First, the DAFP module is essential for enhancement target localization. Its removal caused the largest performance decline and markedly reduced contrast-enhancement scores. Second, the cross-modal attention fusion mechanism plays a critical role in refining the quality of the predicted discrepancy maps. Although this module is lightweight and has little influence on total parameters, MACs, or inference memory, the PSNR reduction caused by replacing it with simple concatenation was greater under the T1 + FLAIR setting (1.034 dB) than under the T1 + T2 setting (0.593 dB), indicating that attention-based feature recalibration becomes increasingly important as the modality gap grows. Third, ControlNet guidance is essential for preserving the structural fidelity of small lesions. Its removal was mainly reflected by reduced VIF values and radiologist-reported degradation of intralesional texture. Overall, the three components play distinct but complementary roles: DAFP is responsible for enhancement target localization, the attention fusion module for cross-modal feature refinement, and ControlNet for spatial constraint and detail preservation.

## 4. Discussion

In this study, we proposed a diffusion-based Difference-Aware Guided Control Network (DAGCN) for synthesizing contrast-enhanced T1-weighted brain MRI from non-contrast T1-weighted images and a water-sensitive auxiliary sequence, either T2-weighted imaging or FLAIR. The proposed framework follows a two-stage design of “discrepancy localization followed by guided generation”. Specifically, a Difference-Aware Fusion and Prediction (DAFP) module first estimates a lesion-related discrepancy map, and this map is then used as an explicit spatial condition in a ControlNet-guided diffusion model to synthesize the final T1-CE image.

Compared with previous CNN- or GAN-based CE-MRI synthesis methods, DAGCN explicitly separates enhancement localization from image generation. Conventional image-to-image synthesis methods generally learn a direct mapping from non-contrast MRI to contrast-enhanced MRI, which may lead to missed subtle enhancement or false-positive enhancement in normal tissue when spatial guidance is insufficient. In contrast, DAGCN provides an interpretable intermediate discrepancy map and uses it to constrain the diffusion generation process. This design may help the model focus on clinically relevant enhancement regions rather than relying solely on global image-to-image transformation.

The quantitative results showed that DAGCN achieved the highest PSNR and NCC under both T1 + T2 and T1 + FLAIR settings, indicating improved intensity fidelity and structural consistency. However, its superiority was not uniform across all metrics. Some baseline methods showed competitive SSIM or VIF values, suggesting that whole-image similarity metrics may not fully reflect lesion-level enhancement fidelity. Therefore, the performance of synthetic T1-CE images should be interpreted together with visual assessment, lesion-focused analysis, ablation results, and representative radiologist-based subjective evaluation. In this regard, DAGCN demonstrated advantages in preserving local lesion enhancement patterns, reducing false-positive enhancement, and maintaining global anatomical plausibility.

The comparison between T2 and FLAIR guidance also provides useful insights. MRI contrast is determined by tissue-specific relaxation properties and sequence parameters, and different pulse sequences emphasize different aspects of tissue contrast [44]. T2-weighted images contain rich water-sensitive information and can provide broad lesion- and edema-related cues. However, cerebrospinal fluid and other nonspecific hyperintense signals may also appear bright on T2-weighted images, which can introduce redundant activations in the predicted discrepancy map. By contrast, FLAIR suppresses cerebrospinal fluid signals while preserving many pathology-related hyperintensities. As a result, FLAIR-guided synthesis produced more spatially focused discrepancy maps, cleaner background suppression, and more specific lesion localization, as observed in the visual analysis. These findings suggest that the choice of auxiliary sequence directly affects the quality of discrepancy-map prediction and subsequent T1-CE synthesis.

Several sources of uncertainty should be considered. First, the quality of the input T1 image is critical because it provides the anatomical baseline for the synthesized T1-CE image. Severe motion artifacts, low signal-to-noise ratio, or intensity inhomogeneity in T1 may be propagated to the synthesized T1-CE image and may also affect discrepancy-map prediction. Therefore, future work should evaluate the robustness of DAGCN under degraded input conditions and incorporate quality-control or uncertainty-estimation mechanisms [45]. Second, although the BraTS 2021 dataset provides co-registered multi-parametric MRI, residual inter-sequence misregistration and intensity normalization differences may affect the construction of the ReLU-based discrepancy target. Third, tumor appearance is heterogeneous across patients, and very small enhancing foci, irregular lesion boundaries, or periventricular abnormalities may challenge the discrepancy predictor. In addition, model hyperparameters, including the empirically selected loss weights and the number of diffusion sampling steps, may influence the balance between global anatomical fidelity and local enhancement accuracy. In the present study, $\lambda_{gen}=10$ and $\lambda_{diff}$ = 1 were selected through limited empirical tuning and stability monitoring rather than a comprehensive sensitivity analysis. Although this setting provided stable convergence in the current experiments, future work should systematically evaluate different loss-weight combinations and their effects on global image fidelity and local enhancement accuracy.

This study has several limitations. First, the experiments were performed on the BraTS 2021 dataset, which mainly contains glioma cases. Therefore, the current findings should not be directly generalized to other enhancing intracranial diseases, such as brain metastases, meningiomas, infection, or inflammatory lesions. Multi-center validation across different tumor types and non-neoplastic enhancing lesions will be necessary before broader clinical application. Potential failure cases should also be considered. Failure was more likely to occur in slices with very small enhancing foci, irregular lesion margins, periventricular abnormalities, low signal-to-noise ratio, or residual motion/misregistration. These cases may challenge the discrepancy predictor and may result in missed subtle enhancement or boundary overestimation. This limitation further motivates future work on 2.5D/3D modeling and uncertainty-aware synthesis [45]. Second, the current DAGCN was implemented as a 2D slice-based framework to reduce memory consumption and enable stable diffusion training with ControlNet guidance. However, this design does not explicitly model through-plane continuity and may lead to inconsistent enhancement across adjacent slices. Future work will investigate 2.5D or 3D diffusion frameworks and latent-diffusion strategies to improve volumetric consistency. Third, the radiologist-based evaluation was performed by only two neuroradiologists and mainly focused on image quality and perceived clinical usefulness. Although the ICC(2,1)-based analysis showed good inter-rater reliability, this cannot fully compensate for the limited number of readers. Therefore, the subjective results should be interpreted as preliminary image quality assessment rather than definitive clinical validation. Importantly, the term “clinical replaceability” in this study refers only to perceived image-level similarity and potential clinical usefulness, not regulatory approval or true clinical interchangeability. DAGCN is currently a research-stage synthesis framework, and we do not recommend using synthesized T1-CE images as a substitute for acquired contrast-enhanced MRI in treatment planning, surgical navigation, radiotherapy planning, treatment-response assessment, or other life-critical clinical decisions. Before clinical deployment, prospective multi-center validation, expanded multi-reader evaluation, predefined diagnostic non-inferiority testing, uncertainty estimation, clinician oversight, and formal regulatory and ethical assessment are required. Therefore, DAGCN should currently be regarded as an investigational tool rather than a clinically approved replacement for standard contrast-enhanced MRI. Fourth, because DAGCN is based on diffusion sampling, the inference time is longer than that of lightweight CNN- or fusion-based methods, which may limit its use in real-time clinical workflows. Under the current 1000-step sampling setting, the estimated sampling time of DAGCN is approximately 47.00 s per slice. For a 50-slice patient volume, this corresponds to approximately 39.2 min without acceleration. Thus, the current implementation is more suitable for offline or semi-automated synthesis, while future work will investigate accelerated sampling strategies, such as DDIM sampling, fewer denoising steps, latent diffusion [43], or model distillation, to improve clinical efficiency.

Another methodological limitation is related to the gradient-detachment strategy used between the discrepancy prediction branch and the diffusion generation branch. Although the Detach() operation stabilizes training and preserves the interpretability of the predicted discrepancy map, it prevents the discrepancy predictor from being directly optimized according to the final synthesis quality. Future work may investigate staged coupled fine-tuning after discrepancy-map convergence, allowing the model to benefit from image-level generation feedback while maintaining stable lesion localization.

Future work will focus on several directions. First, external multi-center validation should be performed to evaluate the robustness of DAGCN across different scanners, imaging protocols, and patient populations. Second, the model should be extended to other enhancing intracranial diseases beyond gliomas to assess its generalizability. Third, 2.5D or 3D diffusion frameworks may be developed to improve volumetric consistency. Fourth, uncertainty-aware synthesis [45] and image quality control mechanisms may help identify unreliable synthetic results. Finally, considering the additional computational cost and longer inference time caused by iterative diffusion sampling, accelerated sampling strategies, such as DDIM sampling, fewer denoising steps, latent diffusion [43], or model distillation, should be explored to improve clinical deployment efficiency.

## 5. Conclusions

In this study, we proposed DAGCN, a difference-aware guided diffusion framework for synthesizing contrast-enhanced T1-weighted MRI from non-contrast T1-weighted images and an auxiliary sequence. The framework first estimates a lesion-related discrepancy map from multi-sequence inputs and then uses this map as spatial guidance in a ControlNet-guided diffusion model. Experiments on the BraTS 2021 dataset demonstrated that DAGCN can generate synthetic T1-CE images with preserved anatomical structure and localized enhancement patterns. Compared with baseline methods, DAGCN achieved the highest PSNR and NCC under both input settings, while showing competitive SSIM and VIF performance. The comparison between auxiliary sequences further indicated that FLAIR guidance provides more specific lesion localization and cleaner suppression of cerebrospinal-fluid-related background interference than T2 guidance. Ablation experiments confirmed that the DAFP module, cross-modal attention fusion, and ControlNet guidance play complementary roles in enhancement localization, feature refinement, and spatially constrained synthesis. These findings suggest that DAGCN may provide a useful technical route for reducing reliance on gadolinium-based contrast administration in selected clinical scenarios. Nevertheless, the current study remains limited by its retrospective design, glioma-focused dataset, and 2D slice-based implementation. Future studies should validate the model on external multi-center cohorts, evaluate diagnostic performance in larger reader studies, improve volumetric consistency, and explore faster diffusion sampling strategies for clinical deployment. The current model remains investigational and should not be used as a replacement for clinically acquired contrast-enhanced MRI or for treatment-planning decisions without further prospective validation and regulatory evaluation.

## Figures and Tables

**Table 1:** Dataset construction and slice-selection summary.

Data Source	Role in This Study	Patient-Level Allocation	Slice-Selection Rule	Samples Used
BraTS 2021 public training set	Model training subset	1000 patients (80%)	Middle 50 axial slices per patient	50,000 paired slices
BraTS 2021 public training set	Internal validation subset	251 patients (20%)	Middle 50 axial slices per patient	12,550 paired slices
BraTS 2021 public validation set	Independent held-out test source	219 patients	Middle 50 axial slices per patient	Held-out source pool
Quantitative test subset from the held-out source	Reported baseline comparison and statistical testing	50 patients (first 50 cases after anonymized-ID sorting)	Middle 50 axial slices per patient	2500 paired test slices per input setting

Note: All splits were performed at the patient level. No slices from the same patient were assigned to more than one subset.

**Table 2:** Quantitative comparison of different synthesis methods under the T1 + T2 and T1 + FLAIR settings.

Model	PSNR	SSIM	VIF	NCC
Algorithm Comparison: Two Inputs (T1 and T2)				
cGAN	14.505 ± 2.243	0.817 ± 0.023	0.451 ± 0.051	0.982 ± 0.008
Hi-Net	14.438 ± 1.592	0.823 ± 0.026	0.473 ± 0.043	0.983 ± 0.008
DDPM	28.441 ± 3.088	0.901 ± 0.022	0.353 ± 0.044	0.976 ± 0.009
U2Fusion	28.628 ± 3.260	0.908 ± 0.023	0.335 ± 0.034	0.977 ± 0.010
DIDFuse	29.040 ± 2.679	0.921 ± 0.017	0.419 ± 0.036	0.982 ± 0.009
SwinFusion	29.766 ± 3.461	0.927 ± 0.021	0.423 ± 0.036	0.983 ± 0.008
DAGCN (ours)	30.011 ± 3.605	0.921 ± 0.017	0.427 ± 0.037	0.986 ± 0.004
Algorithm Comparison: Two Inputs (T1 and FLAIR)				
cGAN	15.284 ± 2.597	0.805 ± 0.023	0.395 ± 0.041	0.978 ± 0.010
Hi-Net	15.296 ± 2.233	0.824 ± 0.028	0.444 ± 0.041	0.980 ± 0.009
DDPM	28.117 ± 2.913	0.892 ± 0.021	0.332 ± 0.039	0.973 ± 0.010
U2Fusion	28.569 ± 3.127	0.907 ± 0.002	0.331 ± 0.033	0.977 ± 0.010
DIDFuse	27.449 ± 2.330	0.899 ± 0.018	0.354 ± 0.041	0.976 ± 0.009
SwinFusion	28.905 ± 3.516	0.919 ± 0.022	0.402 ± 0.034	0.981 ± 0.010
DAGCN (ours)	29.869 ± 3.540	0.919 ± 0.016	0.419 ± 0.036	0.985 ± 0.004

**Table 3:** Quantitative ablation results of the proposed DAGCN framework.

Model	PSNR	SSIM	VIF	NCC
Ablation experiment: Module components (T1 and T2)				
w/o DAFP	27.612 ± 2.714	0.885 ± 0.018	0.325 ± 0.024	0.969 ± 0.012
w/o ATT	29.418 ± 3.362	0.912 ± 0.016	0.394 ± 0.033	0.983 ± 0.005
w/o CLNET	29.465 ± 3.385	0.915 ± 0.016	0.398 ± 0.034	0.983 ± 0.005
DAGCN (ours)	30.011 ± 3.605	0.921 ± 0.017	0.427 ± 0.037	0.986 ± 0.004
Ablation experiment: Module components (T1 and FLAIR)				
w/o DAFP	27.216 ± 2.280	0.879 ± 0.022	0.310 ± 0.037	0.960 ± 0.020
w/o ATT	28.835 ± 2.779	0.907 ± 0.019	0.382 ± 0.046	0.976 ± 0.012
w/o CLNET	28.846 ± 2.799	0.909 ± 0.020	0.386 ± 0.048	0.976 ± 0.013
DAGCN (ours)	29.869 ± 3.540	0.919 ± 0.016	0.419 ± 0.036	0.985 ± 0.004

**Table 4:** Hol-Bonferroni-adjusted patient-level Wilcoxon signed-rank *p*-values comparing DAGCN with baseline methods after averaging slice-level metrics within each patient (*n* = 50).

Setting	Baseline	PSNR	SSIM	VIF	NCC
T1 + T2	cGAN	<0.01	<0.01	<0.01 †	<0.01
T1 + T2	Hi-Net	<0.01	<0.01	<0.01 †	<0.01
T1 + T2	DDPM	<0.01	<0.01	<0.01	<0.01
T1 + T2	U2Fusion	<0.05	<0.05	<0.01	<0.01
T1 + T2	DIDFuse	<0.05	n.s.	<0.05	<0.01
T1 + T2	SwinFusion	<0.05	<0.05 †	<0.05	<0.05
T1 + FLAIR	cGAN	<0.01	<0.01	<0.01	<0.01
T1 + FLAIR	Hi-Net	<0.01	<0.01	<0.01 †	<0.01
T1 + FLAIR	DDPM	<0.01	<0.01	<0.01	<0.01
T1 + FLAIR	U2Fusion	<0.01	<0.01	<0.01	<0.01
T1 + FLAIR	DIDFuse	<0.05	<0.01	<0.01	<0.01
T1 + FLAIR	SwinFusion	<0.05	n.s.	<0.05	<0.01

Note: Values are Holm–Bonferroni-adjusted patient-level Wilcoxon signed-rank *p*-values calculated from per-patient mean metrics (*n* = 50). n.s. indicates no statistically significant difference after correction. † indicates that the corresponding baseline had a higher patient-level mean value than DAGCN; otherwise, significant *p*-values indicate higher patient-level mean values for DAGCN.

**Table 5:** Computational efficiency comparison of DAGCN, baseline methods, and ablation variants.

Model	Params	MACs	Step/Fwd Time	Peak Infer. Mem.
cGAN	1.98 M	4.85 G	3.72 ms	80.43 MB
Hi-Net	3.87 M	13.59 G	5.49 ms	110.66 MB
Diffusion/step	63.15 M	160.27 G	32.64 ms	756.04 MB
U2Fusion	0.66 M	37.94 G	4.90 ms	412.28 MB
DIDFuse	0.26 M	21.51 G	3.65 ms	515.67 MB
SwinFusion	1.47 M	84.05 G	155.16 ms	931.97 MB
DAGCN	92.10 M	212.96 G	47.05 ms	1193.87 MB
DAGCN w/o ATT	92.10 M	212.94 G	46.91 ms	1193.86 MB
DAGCN w/o CLNET	65.13 M	165.15 G	34.56 ms	1010.42 MB
DAGCN w/o DAFP	90.12 M	208.08 G	45.61 ms	1184.66 MB

Note: Params denotes the number of trainable parameters. MACs denotes multiply–accumulate operations measured with an input size of 240 × 240 and a batch size of 1. For cGAN, Hi-Net, U2Fusion, DIDFuse, and SwinFusion, Step/Fwd time indicates the forward inference time. For the vanilla conditional diffusion baseline and DAGCN-based models, Step/Fwd time indicates the single-step denoising time. Peak infer. mem. denotes peak inference GPU memory measured in eval() mode with torch.inference_mode() after warm-up and per-model CUDA cleanup. Complete sampling time depends on the number of denoising steps. Under the current 1000-step sampling setting, the estimated sampling time is approximately 32.64 s per slice for the vanilla conditional diffusion baseline and 47.05 s per slice for DAGCN.

**Table 6:** Summary of radiologist-based subjective scores for representative algorithm-comparison and ablation-study evaluations.

Analysis	Setting	Method	IQ	IC	CE	CF
Algorithm comparison	T1 + T2	U2Fusion	3.52 ± 0.10	3.50 ± 0.00	3.50 ± 0.00	3.50 ± 0.00
Algorithm comparison	T1 + T2	DIDFuse	3.88 ± 0.27	4.03 ± 0.11	3.88 ± 0.27	4.03 ± 0.11
Algorithm comparison	T1 + T2	SwinFusion	3.98 ± 0.28	4.25 ± 0.25	4.19 ± 0.51	4.05 ± 0.15
Algorithm comparison	T1 + T2	DAGCN	4.49 ± 0.08	4.83 ± 0.24	4.55 ± 0.15	4.83 ± 0.24
Algorithm comparison	T1 + FLAIR	U2Fusion	3.60 ± 0.20	3.60 ± 0.20	3.50 ± 0.00	3.50 ± 0.00
Algorithm comparison	T1 + FLAIR	DIDFuse	3.86 ± 0.23	3.86 ± 0.23	4.00 ± 0.00	3.86 ± 0.23
Algorithm comparison	T1 + FLAIR	SwinFusion	4.25 ± 0.29	4.48 ± 0.29	4.82 ± 0.28	4.44 ± 0.16
Algorithm comparison	T1 + FLAIR	DAGCN	4.50 ± 0.00	4.96 ± 0.13	4.57 ± 0.31	4.88 ± 0.21
Ablation study	T1 + T2	w/o DAFP	3.50 ± 0.00	3.50 ± 0.00	3.00 ± 0.00	3.50 ± 0.00
Ablation study	T1 + T2	w/o ATT	4.00 ± 0.00	4.04 ± 0.14	4.00 ± 0.00	4.04 ± 0.14
Ablation study	T1 + T2	w/o CLNET	4.00 ± 0.00	4.04 ± 0.14	4.00 ± 0.00	4.04 ± 0.14
Ablation study	T1 + T2	DAGCN	4.02 ± 0.11	4.13 ± 0.22	4.02 ± 0.11	4.13 ± 0.22
Ablation study	T1 + FLAIR	w/o DAFP	3.50 ± 0.00	3.50 ± 0.00	3.14 ± 0.22	3.50 ± 0.00
Ablation study	T1 + FLAIR	w/o ATT	3.85 ± 0.23	4.03 ± 0.11	4.00 ± 0.00	4.00 ± 0.00
Ablation study	T1 + FLAIR	w/o CLNET	3.92 ± 0.18	4.00 ± 0.00	4.00 ± 0.00	4.00 ± 0.00
Ablation study	T1 + FLAIR	DAGCN	4.22 ± 0.31	4.50 ± 0.00	4.50 ± 0.00	4.50 ± 0.00

Note: Values are presented as mean ± standard deviation. IQ, image quality; IC, clinical replaceability; CE, contrast enhancement; CF, lesion conformity. Scores were assigned by two independent neuroradiologists using a 5-point Likert scale, where higher scores indicate better subjective image quality, stronger clinical usability, more accurate contrast enhancement, or better lesion conformity.

**Table 7:** Inter-rater reliability of the two radiologists’ subjective scores.

Analysis	Setting	IQ	IC	CE	CF	Overall
Algorithm comparison	T1 + T2	0.834 (Good)	0.924 (Excellent)	0.857 (Good)	0.933 (Excellent)	0.894 (Good)
Algorithm comparison	T1 + FLAIR	0.798 (Good)	0.929 (Excellent)	0.919 (Excellent)	0.906 (Excellent)	0.901 (Excellent)
Ablation study	T1 + T2	0.787 (Good)	0.809 (Good)	0.996 (Excellent)	0.807 (Good)	0.893 (Good)
Ablation study	T1 + FLAIR	0.776 (Good)	0.814 (Good)	0.936 (Excellent)	0.815 (Good)	0.858 (Good)
All ratings	All	0.811 (Good)	0.901 (Excellent)	0.930 (Excellent)	0.892 (Good)	0.895 (Good; 95% CI: 0.890–0.901)

Note: ICC values below 0.50, 0.50–0.75, 0.75–0.90, and above 0.90 were interpreted as poor, moderate, good, and excellent reliability, respectively.

## Data Availability

The data analyzed in this study are available from the BraTS 2021 challenge resources, subject to the data access and registration requirements of the dataset organizers.

## Abbreviations

The following abbreviations are used in this manuscript:

CE-MRI	Contrast-enhanced magnetic resonance imaging
GBCA	Gadolinium-based contrast agent
DAGCN	Difference-Aware Guided Control Network
DAFP	Difference-Aware Fusion and Prediction
FLAIR	Fluid-attenuated inversion recovery
PSNR	Peak signal-to-noise ratio
SSIM	Structural similarity index measure
VIF	Visual information fidelity
NCC	Normalized cross-correlation
IQ	Image quality
IC	Clinical replaceability
CE	Contrast enhancement
CF	Lesion conformity
